# The Course of COVID-19 in Patients with Systemic Autoimmune Rheumatic Diseases

**DOI:** 10.3390/jcm11247342

**Published:** 2022-12-10

**Authors:** Marta Rorat, Dorota Zarębska-Michaluk, Justyna Kowalska, Krzysztof Kujawa, Magdalena Rogalska, Dorota Kozielewicz, Beata Lorenc, Katarzyna Sikorska, Piotr Czupryna, Beata Bolewska, Jadwiga Maciukajć, Tomasz Piekoś, Regina Podlasin, Anna Dworzańska, Włodzimierz Mazur, Michał Brzdęk, Anna Szymanek-Pasternak, Robert Flisiak

**Affiliations:** 1 Department of Forensic Medicine, Wroclaw Medical University, 50-367 Wroclaw, Poland; 2 Department of Infectious Diseases, Jan Kochanowski University, 25-369 Kielce, Poland; 3 Department of Adults’ Infectious Diseases, Medical University of Warsaw, 02-091 Warsaw, Poland; 4 Statistical Analysis Centre, Wroclaw Medical University, 50-368 Wroclaw, Poland; 5 Department of Infectious Diseases and Hepatology, Medical University of Białystok, 15-089 Białystok, Poland; 6 Department of Infectious Diseases and Hepatology, Faculty of Medicine, Collegium Medicum in Bydgoszcz, Nicolaus Copernicus University, 87-100 Torun, Poland; 7 Pomeranian Center of Infectious Diseases, Department of Infectious Diseases, 80-210 Gdansk, Poland; 8 Division of Tropical Medicine and Epidemiology, Department of Tropical Medicine and Parasitology, Faculty of Health Sciences, Medical University of Gdańsk, 80–210 Gdańsk, Poland; 9 Division of Tropical and Parasitic Diseases, Department of Tropical Medicine and Parasitology, Faculty of Health Sciences, Medical University of Gdańsk, 80–210 Gdańsk, Poland; 10 Department of Infectious Diseases and Neuroinfections, Medical University of Białystok, 15-089 Białystok, Poland; 11 Department of Infectious Diseases, Poznan University of Medical Sciences, 61-701 Poznan, Poland; 12 Department of Infectious Diseases, District Healthcare Center, 27-200 Starachowice, Poland; 13 Independent Public Healthcare Center in Puławy, Department of Infectious Diseases and Observation for Adults, 24-100 Puławy, Poland; 14 IV-th Department, Hospital for Infectious Diseases, 01-301 Warsaw, Poland; 15 Department of Infectious Diseases and Hepatology, Medical University of Lublin, 20-059 Lublin, Poland; 16 Clinical Department of Infectious Diseases in Chorzów, Medical University of Silesia, 41-500 Katowice, Poland; 17 Department of Infectious Diseases and Hepatology, Wrocław Medical University, 50-367 Wrocław, Poland

**Keywords:** connective tissue diseases, remdesivir, immunodeficiency, respiratory failure

## Abstract

Patients with systemic autoimmune rheumatic disease (SARD) have increased susceptibility to viral infections, including SARS-CoV-2. The aim of this study was to analyse the SARD patient population with COVID-19 (coronavirus disease 2019) in terms of baseline characteristics, severity, course and outcomes of the disease compared with the non-SARD group, and to identify factors associated with prognosis, including remdesivir therapy efficacy. Retrospective study comprised 8220 COVID-19 cases from the SARSTer database, including 185 with SARD. Length of hospitalisation, duration of oxygen therapy, mortality and the need for HFNO (high-flow nasal oxygen) and/or NIV (noninvasive ventilation) were significantly higher in the SARD versus non-SARD group. There was no difference in clinical features on admission to hospital. Patients with SARD were older and more likely to have cardiovascular, pulmonary and chronic kidney diseases. Age, the presence of cardiovascular disease, more severe conditions on admission and higher inflammatory marker values were found to be risk factors for death in the SARD group. In patients with SARD treated with remdesivir, there was a trend towards improved mortality but without statistical significance. Length of hospitalisation, 28-day mortality and the need for HFNO and/or NIV were higher in the SARD group. These patients often had other chronic diseases and were older.

## 1. Introduction

More than 2 years after the World Health Organization (WHO) announced the pandemic “coronavirus disease 2019” (COVID-19) caused by the novel beta coronavirus named “severe acute respiratory syndrome coronavirus 2” (SARS-CoV-2), approximately 500 million people have been infected worldwide, and 6 million of those have died [1]. The virus is mainly transmitted through airborne droplets, but aerosol transmission, transmission through contact with contaminated objects and surfaces and the faecal–oral route have also been documented [2].

The clinical spectrum of SARS-CoV-2 infection is broad, and the severity of the clinical course of the disease varies. It ranges from asymptomatic cases, through mild general and respiratory symptoms, to severe respiratory failure related to bilateral pneumonia. The severity of the course of COVID-19 is determined by the presence of risk factors, including older age, male gender and chronic comorbidities. Obesity, diabetes, malignancies, chronic cardio- and cerebrovascular diseases, chronic pulmonary and liver diseases, kidney failure, immunosuppression and immunodeficiency are listed among independent coexisting health conditions associated with the risk of severe clinical presentation and death [3,4,5].

Since the beginning of the pandemic, patients with SARD (systemic autoimmune rheumatic disease) have also been a focal point in terms of the risk of a severe course of COVID-19 [6]. Due to the altered response of the immune system and immunosuppressive or biological therapies, they are assumed to be a high-risk population. Immunocompromised status resulting from the autoimmune pathogenesis of these diseases and the treatment used results in increased susceptibility to infection by various pathogens. For this reason, the COVID-19 vaccine has been specifically recommended for this group of patients since its introduction. In addition, they have also had priority access to a booster dose [7]. However, the immune response in SARD and COVID-19 is similar. The lung damage caused by the SARS-CoV-2 infection is largely immunologically driven, and the immunomodulatory treatment used for connective tissue diseases protects against cytokine storm aggravation [8].

Data regarding the severity and clinical outcome of COVID-19 in patients with SARD are limited. According to available studies on COVID-19 in patients with and without systemic autoimmune diseases, the risk of infection, hospitalisation rates and risk of a severe course and death seems to be comparable or slightly higher [9]. At the beginning of the COVID-19 pandemic, only supportive therapy was available. However, the rapid global spread of the epidemic drove increased efforts by researchers and scientists to find an antiviral drug for SARS-CoV-2. Analysis of existing drugs’ potential followed by clinical trials confirmed the efficiency of remdesivir, a drug originally developed to combat the Ebola virus, in the treatment of patients with COVID-19 [10]. In fact, by 2022, it was the only drug available with proven antiviral activity against SARS-CoV-2. The effectiveness of remdesivir has subsequently been evaluated in real-world experience, but to date, there has been no study focussing on the SARD patient population [11,12,13].

This retrospective real-world study was designed to:

1. Analyse the SARD patient population with COVID-19 in terms of baseline characteristics, severity, course and outcomes of the disease and compare it with non-SARD group results;
2. Identify factors associated with prognosis;
3. Assess the effect of remdesivir therapy on the course of COVID-19 in patients with SARD.

## 2. Materials and Methods

The analysis included patients whose medical data were obtained from the SARSTer national database, which holds records of 10,308 patients treated between 1 March 2020 and 31 January 2022 in 30 Polish centres. SARSTer is a continuous national real-world experience study tracking treatment outcomes of patients hospitalised in connection with COVID-19. Data were entered retrospectively and submitted online by a web-based platform operated by Tiba sp. z o.o. The project is supported by the Polish Association of Epidemiologists and Infectiologists and Medical Research Agency (grant number ABM-2020/ABM/COVID19/PTEILCHZ). The study was approved by the Ethical Committee of the Medical University of Białystok (29 October 2020, number APK.002.303.2020).

**Figure 1:**
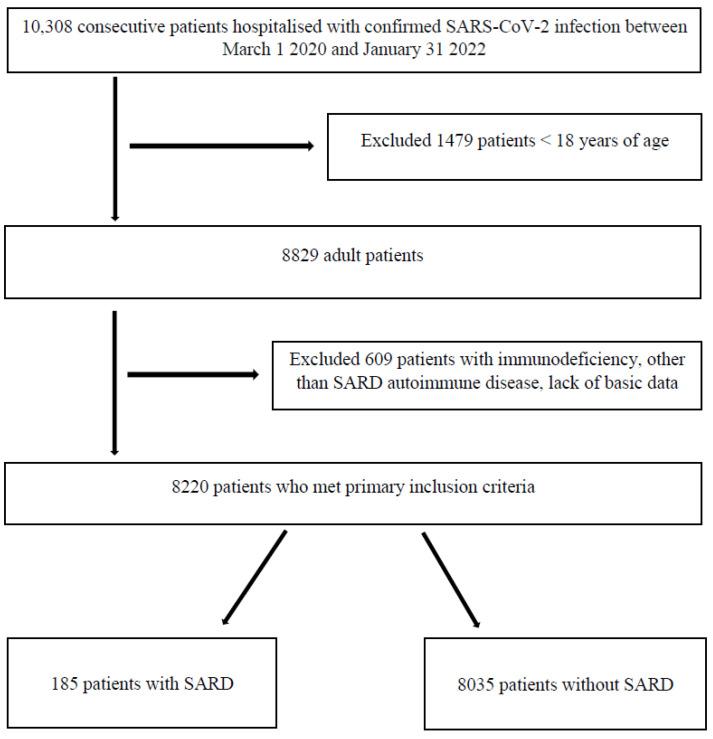
Flowchart of inclusion on the patients. Abbreviations: COVID-19 (coronavirus disease 2019); SARD, systemic autoimmune rheumatic disease.

Patients under 18 years of age were excluded from the study. Among 8829 cases, after the exclusion of post-transplant patients, HIV-infected patients, patients with congenital immunodeficiency and other autoimmune diseases (isolated cases of inflammatory bowel diseases, autoimmune liver diseases, diseases of the central nervous system, psoriasis, diabetes mellitus type 1, glomerulonephritis and idiopathic pulmonary fibrosis), as well as those with missing basic medical data, a group of 185 patients with SARD was selected for retrospective analysis. The remaining 8035 cases were included in the comparison group (Figure 1).

All the patients were diagnosed with SARS-CoV-2 based on positive results of a real-time reverse transcriptase–polymerase chain reaction (RT-PCR) or rapid antigen test using a nasopharyngeal swab specimen. Baseline data included age, gender, body mass index (BMI), comorbidities and medications used to treat them, symptoms of COVID-19, COVID-19 treatment, SpO_2_ (oxygen peripheral blood saturation) and laboratory results: white blood cells count (WBC), platelet count (PLT), C-reactive protein (CRP), procalcitonin (PCT), interleukin 6 (IL-6), ferritin, D-dimers, alanine and aspartate aminotransferases (ALT and AST, respectively), lactate dehydrogenase (LDH) and estimated glomerular filtration rate (eGFR) measured before starting therapy. COVID-19 severity at the time of hospital admission, based on blood oxygen saturation (SpO_2_) and clinical status, was defined using 5 categories: asymptomatic, symptomatic stable with SpO_2_ > 95%, symptomatic unstable with SpO_2_ 91–95%, symptomatic unstable with SpO_2_ ≤ 90% and critically ill with acute respiratory distress syndrome (ARDS). In addition to the baseline characteristics, the primary aim of the study was to compare the severity and outcomes in COVID-19 patients with and without SARD and to identify factors associated with prognosis.

The decisions concerning the treatment regimen were taken entirely by the treating physician based on current knowledge of medication availability and national recommendations. Remdesivir, which until January 2022 was the only drug available with confirmed antiviral activity against SARS-CoV-2, was administered intravenously once daily for 5 days, with a loading dose of 200 mg on day 1, followed by a maintenance dose of 100 mg.

The endpoints of treatment effectiveness were death rate, need for high-flow nasal oxygen (HFNO) therapy or noninvasive ventilation (NIV) and invasive mechanical ventilation (IMV) during 28-day observation.

### Statistical Analysis

The comparison between patient groups was performed with the use of the t-test (for numerical variables) or the t-test with Welch’s correction (for numerical variables when the assumptions of the variance homogeneity have not been met). Nonparametric tests were used for group comparison as appropriate: The chi-square test or the exact Fisher’s test for categorical variables and the Kruskal–Wallis test for continuous variables with non-normal data distribution. The parametric test was applied due to the relatively big sample size, which compensates for the skewed data distribution that occurred in some variables. As central tendency measures, both the mean (with standard deviation SD) and the median (with interquartile range IQR) were used. In the SARD group, patients with and without RDV therapy were compared, and propensity scores were used to reduce selection bias by equating groups based on the following covariates: sex, age, obesity, cardiovascular disease, diabetes, neoplasm, chronic kidney disease, neurological or psychiatric disorders and SpO_2_ (mode—“nearest”) with the use of the R-package “Matchit” [14]. All the tests were two-sided, and a *p*-value less than 0.05 was considered significant. In addition, 95% confidence intervals (CIs) were used to present the statistical analysis results. All analyses concerning SARD subgroups were performed in Statistical Analysis System (SAS) software version 9.4 (Table 1 and Table 2). Other analyses were performed with the use of Statistica v. 13 (Tibco Software Inc. (2017)) and R-environment v. 4.1.2 (R Core team 2021).

## 3. Results

### 3.1. SARD Basic Characteristics

Out of 185 patients included in the analyses, 136 (73.5%) had rheumatoid arthritis, 18 (9.7%) systemic lupus erythematosus (SLE), 5 (2.7%) Sjögren’s syndrome, 4 systemic sclerosis (2.2%) and 22 (11.9%) other SARD of which in 6 (3.2%) cases, at least two SARD were diagnosed. Table 1, Table 2 and Table 3 show demographic and medical data, including baseline laboratory values, for the groups studied.

The effect of chronic immunosuppressive treatment (95/185, 51.4%) on basic patient demographics, saturation (measured on admission), laboratory results, length of hospitalisation, oxygen therapy and death were analysed. Patients who received treatment were more likely to be obese and have a higher CRP concentration and neutrophil count. Immunosuppressive therapies included: glucocorticosteroids, mycophenolate mofetil, cyclosporine, azathioprine, tacrolimus and secukinumab.

### 3.2. SARD versus Non-SARD Comparison

A comparison of the incidence of clinical symptoms (fever, cough, dyspnoea, smell and taste disorders, diarrhoea, vomiting, headache, fatigue/weakness, musculoarticular and bone pain) in both groups was carried out, and no statistical significance was recognised. Only for musculoarticular and bone pain was the *p*-value at 0.059. As for the assessment of mean SpO_2_ and the COVID-19 severity classification at the time of hospital admission, which was based on this parameter, no statistically significant differences were found between the groups with and without SARD. The use of propensity score matching by gender and age somewhat altered the result of the analysis. Among comorbidities, a statistical difference was confirmed with regard to chronic kidney disease and obesity. No significant difference was revealed with regard to duration of oxygen therapy; however, it was confirmed for time to start treatment with dexamethasone (*p* < 0.001).

### 3.3. Remdesivir Treatment in SARD Patients

As shown in Table 4, there were no statistically significant differences in the number of HFNO/NIV, IMV and deaths in the group treated with remdesivir with respect to no treatment. The analysis also failed to demonstrate the efficacy of remdesivir therapy in the group of patients in whom treatment was initiated up to 7 days after the onset of symptoms: HFNO/NIV *p* = 0.620, IMV *p* = 0.234, death *p* = 0.495. However, the effectiveness of the therapy was demonstrated in the group of patients without SARD, both in terms of the frequency of HFNO/NIV (*p* < 0.001), IMV use (*p* < 0.001) and reduction in death (*p* < 0.001).

Propensity score matching was used to evaluate the effectiveness of RDV therapy on COVID-19 clinical course in SARD patients (Table 5). A group of 56 people was selected from among 129 patients not treated with remdesivir. There were no statistically significant differences between these groups in the frequency of HFNO/NIV, IMV and deaths. The survival probability during stay in hospital did not differ between the two compared groups (log-rank test: chi-sq. = 0.756, *p* = 0.449).

## 4. Discussion

In our study, among patients with SARD, rheumatoid arthritis cases predominated (73.5%), and half of patients were on immunosuppressive treatment. The mean age was 66.9 years; 65.9% of patients had been diagnosed with cardiovascular disease, 20% were obese and 18.4% had diabetes. More than 70% of the group had SpO_2_ < 95% on admission to the hospital. The most common abnormalities found in laboratory tests included elevated inflammatory markers, lymphopenia, elevated D-dimer levels and LDH activity, as well as reduced GFR. Patients who died, compared with those who survived, were older, had worse base respiratory capacity, higher inflammatory parameters and lower GFR (Table 1 and Table 2).

We found no differences in clinical characteristics, including lab test results, between the SARD and non-SARD groups (Table 3). The initial condition of patients on admission to hospital was similar. Patients with SARD were more likely to have cardiovascular, pulmonary, and chronic kidney diseases, and the mean age was higher. Both hospitalisation length, duration of oxygen therapy, the need for HFNO (high-flow nasal oxygen) and/or NIV (noninvasive ventilation) and mortality were significantly higher in the SARD group. Such an observation was not made regarding the need for intubation because patients with a poor prognosis due to age, disease burden or severe COVID-19 complications were frequently disqualified from further treatment.

In this study, 56 of 185 patients with SARD received RDV (remdesivir), and the mortality rate in this group was lower compared with those who were not treated with RDV (16.1% vs. 25.6%) (Table 4). However, the difference was not statistically significant, *p* = 0.156. In addition, propensity score matching did not change the results, *p* = 0.341 (Table 5).

Data on the impact of systemic autoimmune rheumatic diseases on the risk of SARS-CoV-2 infection and its course are inconclusive. Zen et al. analysed 916 cases of autoimmune rheumatic diseases, among which patients with SLE (systemic lupus erythematosus) were dominant (397/916), and of whom only 148 patients had symptoms. The clinical course in the study group was mild, and no deaths occurred. The authors concluded that the incidence of COVID-19 appears to be similar to the general population [15]. Similar observations came from other works [16,17,18]. However, an analysis by Akiyama et al. of 62 observational studies involving 319,025 patients with autoimmune diseases showed that the incidence of COVID-19 was higher than in the control group (highest in the autoimmune liver disease and SLE/SjS/SSc group 0.034, 60% were treated with glucocorticoids in this subgroup) [19]. According to all available data, the prevalence of COVID-19 in patients with systemic autoimmune rheumatic diseases is expected to be slightly higher than in the general population [9,20].

The course of COVID-19 in systemic autoimmune rheumatic diseases patients also does not appear to differ significantly from the general population [9], although the likelihood of adverse outcomes is undoubtedly influenced by well-known risk factors for severe disease (age, gender, comorbidities) and autoimmune disease treatment [9].

In one of the first reports, D’Silva et al. demonstrated that patients with selected rheumatic diseases (RD), as opposed to those without rheumatic disease, were more likely to require admission to intensive care and mechanical ventilation (OR 3.22, 95% CI 1.16–8.92, *p* = 0.02). In general, there were no statistical differences in clinical features (symptoms, laboratory results and comorbidities), oxygen requirements, hospitalisation and death rates. Only a higher white blood cell count and a higher incidence of coronary artery disease were found in the group with RD [21]. In the study by Pablos et al., increasing age, male gender and having an autoimmune rheumatic disease (OR 1.82, 95% CI 1.00–3.30) were found to be independent factors associated with COVID-19 severity. Obesity and cardiovascular disease were more common in the group of patients with RD. However, there were no significant differences during COVID-19, including serious complications, oxygen support, laboratory results, intensive care unit admission and death [22]. Qi et al. reached similar conclusions after analysing their own data, although a meta-analysis of five studies performed by these researchers revealed a higher risk of an adverse course in patients with rheumatic diseases (RR = 1.70, 95% CI 1.35–2.13, *p* < 0.0001, I 2 = 53%) [23].

Analyses conducted on large groups of patients in the United States and South Korea demonstrated a slightly higher risk of death in COVID-19 patients with RD [24,25]. A Danish-population-based study of 58,052 people with RD showed that the incidence of hospitalisation was higher in this group compared to the general population (HR 1.46, 95% CI 1.15–1.86), especially in rheumatoid arthritis (RA) and vascular disease patients. The risk of severe disease was also higher in hospitalised patients (HR 1.43, 95% CI 0.80–2.53), although the small group of patients with RD makes inference difficult [26]. In a study by Williamson et al. conducted on behalf of the NHS (National Health Service) England, the analysis of data from more than 17 million adults from the OpenSAFELY platform showed that autoimmune diseases (rheumatoid arthritis, lupus or psoriasis) were associated with a higher risk of death but that the association was not as strong when compared with other chronic diseases (HR 1.19, 95% CI 1.11–1.27) [27]. A meta-analysis of 65 observational studies by Akiyama et al. including 2,766 patients showed that the rate of hospitalisation due to COVID-19 was 0.35; this was highest in RD (0.54) and AHD (0.52) [19]. In addition, the mean age (58.3 years) and prevalence of underlying comorbidities (71.8%) were highest in the RD group. Mortality across the entire study group was 0.066, and the highest was in RD (0.097) and AHD (0.094). Age and comorbidities were found to be poor predictors of COVID-19 in AD.

Chronic treatment is an important factor influencing the prognosis of patients with RD. Akiyama et al. demonstrated that glucocorticoids, conventional synthetic disease-modifying antirheumatic drugs (csDMARDs) and the combination of this therapy with biological (b) or targeted synthetic (ts) DMARDs increased the risk of negative outcomes. The risk of hospitalisation and death was lower in those treated with b/tsDMARDs monotherapy, particularly antitumour necrosis factor agents [19]. These findings are partly consistent with an analysis from the COVID-19 Global Rheumatology Alliance registry, including 600 cases from 40 countries [28], in which a prednisone dose of ≥10 mg/day was associated with a higher likelihood of hospitalisation (OR 2.05, 95% CI 1.06 to 3.96). Such a correlation was not observed regarding the use of DMARDs alone or in combination with biologics/Janus Kinase inhibitors, nonsteroidal anti-inflammatory drugs (NSAIDs) and antimalarial drugs. Patients treated with anti-TNF had a reduced risk of hospitalisation (OR 0.40, 95% CI 0.19 to 0.81). In the study group, the median age of hospitalised patients was 62 years, with a group of patients with RD (38%) and SLE (17%) dominating; significantly, 81% were in remission or presented minimal/low disease activity. In our analysis, the risk of death was not higher in patients taking immunosuppressive drugs, including GC, which may be due to the small study group and lack of data on doses used, making it impossible to divide patients into subgroups according to this parameter. The risk factors for death included age, the presence of cardiovascular disease, status on admission and laboratory results (in particular, higher values of inflammatory markers), of which the influence on death has been confirmed in numerous publications [4,22,29,30].

Our results are mostly consistent with those of other researchers. Some discrepancies between researchers may be caused by different methodologies and, above all, by the differences in groups studied in terms of the type of rheumatic diseases and by the chronic treatment used.

The therapeutic management of patients with RD and COVID-19 depends on the severity of the baseline patient’s condition, oxygen requirements and phase of the disease and does not generally differ from that of patients without RD [7,31]. In this study, the mortality rate in patients treated with RDV group was lower compared with those who were not treated; however, it did not reach statistical significance. RDV was initiated on average at 6.3 days from the onset of symptoms, although it should be noted that in patients receiving immunosuppressive treatment, an even longer period from the onset of the disease to the administration of the drug is permitted due to longer viral replication [32].

Although no results, to the best of our knowledge, are available on the use of RDV exclusively in patients with RD, our observations of a trend towards lower mortality support the results of clinical trials and RWE studies conducted in the general population on patients with COVID-19 [10,11,12,13]. The small group size may be a limiting factor in the statistical power of the comparative analysis.

The final outcome of patients with COVID-19 and SARD depends on a number of mutually influential factors. Undoubtedly, the concomitant use of different therapies might have a significant impact. More than half of the patients in this study (95/185, 51.4%) required immunosuppressive treatment due to the progression of COVID-19 to the cytokine storm phase; dexamethasone was used in 95 patients, 23 of whom also received tocilizumab. In the treatment of the hyperinflammatory state, dexamethasone can be used in patients with RD, even those receiving other immunosuppressive therapies, while the addition of a second immunomodulator requires individual consideration based on the immunosuppression used for RD [20]. The percentage of patients with RD treated with dexamethasone was significantly higher among those who died compared with survivors (*p* = 0.002).

We are aware of the limitations of the current analysis. This includes possible bias associated with the retrospective observational nature of the study. Although many covariates were adjusted in the PSM analysis, some confounding variables may have remained. The low number of patients in the group with SARD significantly limits the power of this study, the range of statistical analysis and its conclusions. In addition, we did not capture data on vaccination status, SARS-CoV-2 variants, the activity of the rheumatic diseases, the correlation between RD and other chronic diseases, the duration of immunosuppression and doses of drugs used before and at the time of hospital admission due to COVID-19. Although the use of biological therapies in the treatment of autoimmune diseases, including RD, is on the rise, none of the patients in the analysed group had been treated with tocilizumab for autoimmune disease. Therefore, we could not analyse the effect of long-term tocilizumab therapy on the severity of COVID-19, even though it would be expected that such type of immunosuppression may play a protective role in the development of an excessive immune-mediated inflammatory response associated with SARS-CoV-2 infection [33]. Finally, the SARD group was heterogeneous in terms of the type of rheumatic disease and its treatment, status on admission and the time to the start of antiviral treatment. However, the key strength of our analysis was the collection of data from the real-world population from different parts of the country, which allows us to generalise the results.

## 5. Conclusions

Length of hospitalisation, 28-day mortality as well as the need for HFNO and/or NIV were higher in the SARD group compared with patients without SARD. These patients were more likely to suffer from other chronic diseases and be older. The risk factors for death included older age, the presence of cardiovascular disease, more severe conditions on admission and higher values of inflammatory markers. In the analysed group of patients, remdesivir therapy did not benefit patients with SARD.

## Figures and Tables

**Table 1:** Basic demographic and medical data in the group of patients with SARD.

Baseline Characteristic	All *n* = 185	Survived *n* = 143	Deceased *n* = 42	*p*-Value
Female gender, *n* (%)	137 (74.1)	107 (74.8)	30 (71.4)	0.691
Age in years, mean (SD); median (IQR)	66.9 (13.3); 69 (59–76)	65 (13.7); 66 (56–75)	73.3 (9.3); 73 (69–80)	<0.001
BMI, mean (SD); median (IQR)	26.8 (5); 26.1 (23.4–30.5)	27 (5); 26.2 (24–30.5)	26.2 (4.7); 25.8 (23–28.1)	0.473
Concomitant diseases, *n* (%)				
Cardiovascular disease	122 (65.9)	85 (59.4)	37 (88.1)	<0.001
Respiratory disease	27 (14.6)	22 (15.4)	5 (11.9)	0.804
Diabetes	34 (18.4)	25 (17.5)	9 (21.4)	0.651
Malignant neoplasm	10 (5.4)	7 (4.9)	3 (7.1)	0.697
Chronic kidney disease	14 (7.6)	10 (7)	4 (9.5)	0.525
Obesity	37 (20)	32 (22.4)	5 (11.9)	0.136
Rheumatoid arthritis, *n* (%)	136 (73.5)	106 (74.1)	30 (71.4)	0.843
Immunosuppressive treatment, *n* (%)	95 (51.4)	71 (49.6)	24 (57.1)	0.483
Methotrexate	35 (18.9)	32 (22.4)	3 (7.1)	0.026
Corticosteroids	62 (33.5)	45 (31.5)	17 (40.5)	0.353
SpO_2_ in % on hospital admission, mean (SD); median (IQR)	89.7 (7); 91 (87–94)	90.8 (5.8); 92 (88–94)	85.8 (9.1); 88 (85–93)	<0.001
Classification on hospital admission, *n* (%):				<0.001
Asymptomatic	3 (1.6)	2 (1.4)	1 (2.4)	
Symptomatic stable with SpO_2_ > 95%	50 (27)	44 (30.8)	6 (14.3)	
Symptomatic unstable with SpO_2_ ≤ 95%	63 (34.1)	56 (39.1)	7 (16.7)	
Symptomatic unstable with SpO_2_ ≤ 90%	65 (35.1)	40 (28)	25 (59.5)	
ARDS	4 (2.2)	1 (0.7)	3 (7.1)	
Remdesivir, *n* (%)	56 (30.3)	47 (32.9)	9 (21.4)	0.181
Dexamethasone, *n* (%)	95 (51.4)	65 (45.4)	30 (73.2)	0.002
Tocilizumab, *n* (%)	23 (12.4)	14 (9.8)	9 (21.9)	0.058

Abbreviations: SARD, systemic autoimmune rheumatic disease; SD, standard deviation; IQR, interquartile range; BMI, body mass index; SpO_2_, oxygen saturation; ARDS, acute respiratory distress syndrome.

**Table 2:** Laboratory characteristics in patients with SARD, mean (SD); median (IQR).

Characteristic	All *n* = 185	Survived *n* = 143	Deceased *n* = 42	*p*-Value
CRP (mg/L)	84.8 (73.4); 66.1 (22.8–139.5)	72.8 (64.8); (17.6–118.1)	126.9 (86.4); 109.5 (74.1–146.5)	<0.001
Procalcitonin (ng/mL)	1.5 (14.2); 0.1 (0.1–0.2)	0.19 (0.54); 0.07 (0.05–0.13)	5.12 (27.5); 0.3 (0.15–0.57)	<0.001
WBC (/uL)	7146.4 (14.2); 6150 (4600–8700)	6660.0 (4016.7); 5885 (4360–7635)	8766.2 (4256.6); 8345 (6100–10590)	<0.001
Lymphocytes (/uL)	1085.9 (680.8); 950 (600–1360)	1057.1 (660.4); 920 (600–1335)	1209.0 (761.5); 1005 (700–1500)	0.301
Neutrocytes (/uL)	5374.7 (3744.8); 4145 (3050–6480)	4919 (3522.1); 3980 (2855–5465)	7319.3 (4094.4); 6420 (4140–8450)	<0.001
PLT (/uL)	217,714.3 (94,330.7); 195,000 (154,000–277,000)	222,612.7 (98,117.8); 198,500 (154,000–279,000)	200,325.0 (78,063.6); 186,500 (149,500–245,000)	0.285
D-dimers (ug/mL)	2222.6 (5691.3); 968.5 (680–1610)	2255.4 (6226.3); 921 (670–1457)	2090.8 (2660.2); 1200 (730–2325)	0.163
eGFR < 60 mL/min/m^2^, *n* (%)	42 (22.7)	26 (18.2)	16 (39.1)	0.001
LDH (IU/L)	445 (266.3); 375 (287–529)	394.1 (230.4); 319 (268–453)	599.1 (337.9); 492 (424–668)	<0.001
ALT (IU/L)	33.8 (27.9); 24 (17–42)	32 (26.7); 23 (16–39)	40.4 (31.4); 30 (23–51)	0.018
AST (IU/L)	54.5 (46.7); 43 (30–60)	47.4 (41.9); 38 (25–55)	75.5 (54.0); 60 (40–103)	<0.001

Abbreviations: CRP, C-reactive protein; WBC, white blood cell; PLT, platelet count; IL-6, interleukin 6; eGFR, estimated glomerular filtration rate; LDH, lactate dehydrogenase; ALT, alanine transaminase; AST, aspartate aminotransferase.

**Table 4:** Impact of remdesivir therapy on the course of COVID-19 in patients with SARD.

		RDV *n* = 56	No RDV *n* = 129	*p*-Value
HFNO/NIV	No	46 (82.1%)	102 (79.1%)	0.631
	Yes	10 (17.9%)	27 (20.9%)	
IMV	No	48 (85.7%)	119 (92.2%)	0.852
	Yes	8 (14.3%)	10 (7.8%)	
Death	No	47 (83.9%)	96 (74.4%)	0.156
	Yes	9 (16.1%)	33 (25.6%)	

Abbreviations: RDV, remdesivir; HFNO, high-flow nasal oxygen; NIV, noninvasive ventilation; IMV, invasive mechanical ventilation.

**Table 5:** Impact of remdesivir therapy on the course of COVID-19 in patients with SARD after PSM.

		RDV *n* = 56	No RDV *n* = 56	*p*-Value
HFNO/NIV	No	46 (51.1%)	44 (48.9%)	0.634
	Yes	10 (45.5%)	12 (54.6%)	
IMV	No	48 (48.5%)	51 (51.5%)	0.376
	Yes	8 (61.5%)	5 (38.5%)	
Death	No	47 (52.2%)	43 (47.8%)	0.341
	Yes	9 (40.9%)	13 (59.1%)	

Abbreviations: PSM, propensity score matching.

**Table 3:** Baseline clinical and laboratory characteristics in the groups with and without SARD.

Characteristic	SARD *n* = 185	Non-SARD *n* = 8035	*p*-Value
Gender			
Female, *n* (%)	137 (74.1)	3690 (45.9)	<0.001
Age (y), mean (SD), median (IQR)	66.9 (13.3); 69 (59–76)	62.8 (16.8); 64 (51–75)	<0.001
Concomitant diseases, n (%)	122 (65.9)	4602 (57.3)	0.018
Cardiovascular diseases	27 (14.6)	740 (9.2)	0.013
Pulmonary diseases	10 (5.4)	564 (7)	0.394
Malignant neoplasm	37 (20)	2184 (27.2)	0.030
Obesity	34 (18.4)	1658 (20.6)	0.453
Diabetes	14 (7.6)	354 (4.4)	0.040
Chronic kidney disease			
Neurological and psychiatric disorders	14 (7.6)	810 (10.1)	0.260
SpO_2_ on hospital admission (%), mean (SD), median (IQR)	89.7 (7); 91 (87–94)	90.2 (6.9); 92 (88–95)	0.308
Duration of oxygen therapy (days), mean (SD); median (IQR)	8.4 (8.5); 7 (1.5–12)	7.1 (8.7); 6 (0–10)	0.047
Length of hospitalisation (days), mean (SD); median (IQR)	14.6 (9.6); 12 (9–18)	12.5 (8); 11 (8–15)	0.003
Death, *n* (%)	42 (22.7)	1155 (14.4)	0.002
Laboratory test results, mean (SD); median (IQR)			
CRP (mg/L)	84.8 (73.4); 66.1 (22.8–129.4)	81.4 (77.7); 59.5 (21–120.3)	0.559
Procalcitonin (ng/mL)	1.5 (14.2); 0.1 (0.1–0.2)	0.8 (7.9); 0.1 (0.1–0.2)	0.318
WBC (/uL)	7146.4 (14.2); 6150 (4600–8700)	6992.9 (4674.9); 6000 (4500–8210)	0.664
Lymphocytes (/uL)	1085.9 (680.8); 950 (600–1360)	1229.7 (2374.9); 1000 (700–1400)	0.447
Neutrocytes (/uL)	5374.7 (3744.8); 4145 (3050–6480)	5241.5 (10396.2); 4300 (2900–6400)	0.872
PLT (/uL)	217,714.3 (94,330.7); 195,000 (154,000–277,000)	207,416.8 (99,902.1); 192,000 (145,000–254,000)	0.169
IL-6 (pg/mL)	69.4 (78.5); 40.5 (16.8–96.7)	87 (305.6); 37.8 (14.6–80)	0.553
D-dimers (ug/mL)	2222.6 (5691.3); 968.5 (680–1610)	2148.3 (6444.7); 846.1 (500–1551)	0.883
eGFR < 60 mL/min/m^2^, *n* (%)	42 (22.7)	1725 (21.5)	0.624
LDH (IU/L)	445 (266.3); 375 (287–529)	422.1 (273.1); 369 (268–501.5)	0.417
ALT (IU/L)	33.8 (27.9); 24 (17–42)	46.6 (98.4); 31.2 (21–51)	0.082
AST (IU/L)	54.5 (46.7); 43 (30–60)	69.9 (546.3); 42 (30–63)	0.762
Treatment mean (SD), median (IQR)			
Remdesivir—time to start of treatment (days)			
From onset of symptoms	6.3 (3.5); 6.5 (3–8.5)	6.3 (3.3); 6 (4–8)	0.980
From diagnosis	2.3 (2.6); 1 (1–3)	2.3 (2.6); 1 (1–3)	0.928
Tocilizumab—time to start of treatment (days)			
From onset of symptoms	9.6 (5.9); 7 (5–14)	9.2 (4.7); 9 (7–12)	0.692
From diagnosis	5.2 (5.2); 3 (2–6)	4.6 (3.5); 4 )2–7)	0.407
Dexamethasone—time to start of treatment (days)			
From onset of symptoms	7.5 (4); 7 (5–10)	7.8 (4.4); 8 (5–10)	0.520
From diagnosis	2.4 (2.7); 1 (1–4)	3 (3.3); 1 (1–5)	0.073
HFNO/NIV, *n* (%)	37 (20)	903 (11.2)	<0.001
IMV, *n* (%)	18 (9.7)	563 (7)	0.153

Abbreviations: SARD, systemic autoimmune rheumatic disease; SD, standard deviation; IQR, interquartile range; SpO_2_, oxygen saturation; CRP, C-reactive protein; WBC, white blood cell; PLT, platelet count; IL-6, interleukin 6; eGFR, estimated glomerular filtration rate; LDH, lactate dehydrogenase; ALT, alanine transaminase; AST, aspartate aminotransferase; HFNO, high-flow nasal oxygen; NIV, noninvasive ventilation; IMV, invasive mechanical ventilation.

## Data Availability

The datasets used and analysed during the current study are available from the corresponding author upon reasonable request.
